# Comparison of electroencephalogram between propofol- and thiopental-induced anesthesia for awareness risk in pregnant women

**DOI:** 10.1038/s41598-020-62999-5

**Published:** 2020-04-10

**Authors:** Hee-Sun Park, Yeon-Su Kim, Sung-Hoon Kim, A-Rom Jeon, Seong-Eun Kim, Woo-Jong Choi

**Affiliations:** 1 Department of Anesthesiology and Pain Medicine, University of Ulsan College of Medicine, Asan Medical Center, Seoul, Korea; 2 Department of Electronics and Control Engineering, Hanbat National University, Daejeon, Korea

**Keywords:** Neuroscience, Medical research

## Abstract

There have been few comparative studies using electroencephalogram (EEG) spectral characteristics during the induction of general anesthesia for cesarean section. This retrospective study investigated the differences in the depth of anesthesia through EEG analysis between propofol- and thiopental-induced anesthesia. We reviewed data of 42 patients undergoing cesarean section who received either thiopental (5 mg/kg) or propofol (2 mg/kg). EEG data were extracted from the bispectral index (BIS) monitor, and 10-second segments were selected from the following sections: 1) Stage I, BIS below 60 after induction; 2) Stage II, after intubation completion; 3) Stage III, end-tidal sevoflurane above 0 vol%. The risk of awareness was represented by the BIS and entropy measures. In Stage III, the thiopental group (n = 20) showed significantly higher BIS value than the propofol group (n = 22) (67.9 [18.66] vs 44.5 [20.63], respectively, *p* = 0.002). The thiopental group had decreased slow-delta oscillations and increased beta-oscillations as compared to the propofol group in Stages II and III (*p* < 0.05). BIS, spectral entropy, and Renyi permutation entropy were also higher in the thiopental group at Stages II and III (*p* < 0.05). In conclusion, frontal spectral EEG analysis demonstrated that propofol induction maintained a deeper anesthesia than thiopental in pregnant women.

## Introduction

Neuraxial anesthesia is a preferred form of anesthesia for cesarean section^1^. The rate of general anesthesia for cesarean section has been relatively low and has been declining in tertiary care hospitals^2^. However, general anesthesia is still required in emergencies or when regional anesthesia is contraindicated, such as in the presence of a coagulation abnormality or patient refusal. Cesarean section under general anesthesia is associated with a high risk of maternal intraoperative awareness. The fifth National Audit Project (NAP5) reported the incidence of accidental awareness during the cesarean section under general anesthesia to be approximately 1:670 (1:380–1300)^3^. General anesthesia for cesarean section is traditionally induced with thiopental and succinylcholine using a rapid sequence induction (RSI) method. Although propofol has most commonly been used in cesarean section as an induction agent, still some anesthesiologists continue to prefer thiopental for obstetric anesthesia for historical and traditional reasons^4,5^.

The bispectral index (BIS) monitor, which processes the frontal electroencephalogram (EEG) signal to generate a number between 0 (deep anesthesia) and 100 (awake), is used to measure the hypnotic component of anesthesia. A range of 40–60 is considered to be suitable for general anesthesia. When BIS is over 60, there is the possibility of awareness risk during general anesthesia. BIS monitoring has been reported to reduce the risk of intraoperative awareness in populations at high risk^6,7^. A previous study reported that for patients receiving 4 mg/kg of thiopental, the BIS value is >60 at the time of incision^8^. Parturients receiving 2–2.5 mg/kg of propofol present with a lower BIS after endotracheal intubation and skin incision as compared to those receiving thiopental^9,10^.

Some studies have suggested that EEG oscillations are related to altered states of consciousness produced by anesthetics^11–15^. However, there is a lack of detailed characterization of oscillatory EEG activity regarding thiopental and propofol administration in pregnant women. Therefore, characterizing EEG spectral dynamics should help to establish the risk of awareness during general anesthesia. We aimed to investigate whether there are any differences in frontal brain oscillations between propofol- and thiopental-induced general anesthesia for cesarean section. Further, BIS and entropy analyses were also conducted to evaluate awareness risk in each group.

## Methods

### Patient population

This study was approved by the Asan Medical Centre institutional review board (IRB number 2019–0737). The need for informed consent was waived, considering the retrospective nature of this analysis. The study conformed to the tenets of the Declaration of Helsinki.

A retrospective analysis of EEG data recorded during elective cesarean section was conducted between September 2017 and May 2019. Our institute has traditionally used only thiopental (Pentothal sodium, JW Pharmaceutical, Seoul, Korea) for cesarean section, but there was a temporary supply interruption from November 2017 until March 2018. Propofol was then used as an alternative induction agent for cesarean section. After the re-supply of thiopental, agent selection was decided by the attending anesthesiologist.

We identified all parturient women who received general anesthesia. Through chart review, parturients were divided into two groups according to the anesthetic agent: the thiopental and propofol groups. Inclusion criteria were between 20 and 44 years old, American Society of Anesthesiologist grade 1 or 2, and elective operation. Patients with the following conditions were excluded from the study: (1) cases where regional anesthesia was converted to general anesthesia; (2) exposure to sevoflurane before the induction of general anesthesia; (3) missing Vital Recorder data; (4) difficult airway management cases.

#### Anesthetic management

Our institutional standardized anesthesia protocols were used for cesarean section. All parturient women received at least 3 min of pre-oxygenation using 100% oxygen, followed by RSI with cricoid pressure. Thiopental 5 mg/kg or propofol 2 mg/kg was administered, followed by succinylcholine 1.5 mg/kg to facilitate intubation. After disappearance of fasciculation and of the electromyography (EMG) activity bar on the BIS monitor, all patients were intubated with a 6.5-cuffed tracheal tube using a video-scope. After confirming successful intubation, rocuronium 0.5 mg/kg was immediately administered to achieve further muscle relaxation. Anesthesia was maintained with 50% nitrous oxide (N_2_O) in oxygen (6 L/min) and sevoflurane. The initially inspired concentration of sevoflurane was set at 2.0 vol% on the vaporizer until the end-tidal sevoflurane (Et-Sevo) reached 1.3 vol%. A skin incision was made after the anesthesiologist confirmed intubation. After delivery, intravenous midazolam 0.03–0.05 mg/kg and fentanyl 100 *μg* were administered. The concentration of sevoflurane was reduced to 1.0 vol% with a flow of 2 L/min. Additive intravenous midazolam was injected to maintain the BIS value at <60. Intravenous patient-controlled analgesia (IV-PCA) was initiated during surgery. Sevoflurane and N_2_O were discontinued after skin closure, and any residual neuromuscular block was reversed using glycopyrrolate 0.4 mg and pyridostigmine 15 mg. Neonatal Apgar scores at 1 and 5 min were assessed by the pediatrician and nurse in charge of each neonate. A clinical nurse specialist, who was responsible for the postoperative pain control, evaluated dreaming, nightmares and any other experiences related to awareness in parturients after the operation and notified the anesthesiologists of any issue.

### Data acquisition

BIS was measured continuously using a BIS monitor (VISTA_,_ Aspect Medical Systems, Norwood, MA, USA). A BIS Quatro (Medtronic, Minneapolis, MN, USA) four-electrode sensor was placed on the patient’s forehead. The vital signs, ventilator values, patient monitor values, and BIS and raw EEG data were recorded in the automatic recording program, Vital Recorder^16^. Our institute has recorded certain events during surgery such as intravenous agent administration, and birth on Vital Recorder since 2017. BIS and EEG data were stored at a rate of 125 Hz. Et-Sevo, N_2_O and end-tidal carbon dioxide (EtCO_2_) were continuously monitored on the ventilator using the gas analyzer on the patient monitor. We defined the following segments by examining the Vital Recorder data: (1) Awake, (pre-anesthesia); (2) Stage I, the first time period where BIS was below 60 after the agent administration; (3) Stage II, immediately after intubation completion when the first EtCO_2_ flow appeared on the ventilator monitor (4) Stage III, the first period when Et-Sevo was above 0 vol% after intubation completion. The following data were also reviewed: maternal demographic data, indication for general anesthesia, and the induction agent dosage.

### EEG analysis

#### Data pre-processing

Pre-processing of the data was completed using MATLAB (MathWorks, Natick, MA, USA). We applied a low-pass filter designed with a low cut-off frequency of 0.5 Hz and a high cut-off frequency of 50 Hz. EEG dynamics were analyzed at four distinct periods: (1) Awake, (2) Stage I, (3) Stage II, and (4) Stage III. For each subject, a 10-s EEG segment was selected from the artifact-free EEG data, where motion or electrocautery artifacts were not present.

#### Time-frequency analysis

The power spectral density measures the frequency distribution of power within a signal, where power is the 10 * log10 of the EEG signal amplitude squared. The EEG power spectrum quantifies the energy in the EEG signal at each frequency, and the EEG spectrogram displays the EEG power spectrum as a function of time. In these spectrograms, frequencies were arranged along the y-axis and time along the x-axis. Power is indicated using color on the decibel scale. Spectral analysis of activity was performed with multitaper methods and implemented in the Chronux toolbox^15^. The multitaper parameters were selected as follows: window lengths of T = 2 s with a 1.9 s overlap, time-bandwidth product TW = 2, and the number of tapers K = 3.

We set up three groups: Stage I, II and III. We computed group-averaged spectrograms by taking the median power across all subjects at each time and frequency of each stage. The group-averaged spectra were calculated by taking the median power of individual spectrograms at each frequency across the entire 10-second epoch, and median power at each frequency was calculated across subjects for each group. The interquartile range (IQR) was displayed as a shaded area on the averaged spectra.

#### Awareness analysis

We assessed the risk of awareness during general anesthesia using BIS, spectral entropy, and Renyi permutation entropy (RPE). Spectral entropy and RPE were computed using raw EEG data extracted from the BIS monitor. The spectral entropy is a measure of the signal’s spectral power distribution based on Shannon entropy. The greater the spectral entropy is, the more uniform is the power spectral distribution. In this way, spectral entropy can quantify the regularity of the power spectrum and has been used to assess anesthetic drug effects^17^.

The RPE has been suggested as the best index to measure the depth of anesthesia^18^, so we applied this to our EEG data. The RPE is a type of permutation entropy based on Renyi entropy and is given by

$$RPE=\frac{\log \,{\sum }_{j=1}^{m!}{p}_{j}^{\alpha }}{(1-\alpha )\mathrm{ln}m}$$

where *p*_*j*_ is the probability of the *j*_*th*_ permutation occurring, ${p}_{j}=\frac{{n}_{j}}{{\sum }_{j=1}^{m!}{n}_{j}}$, and *n*_*j*_ is the number of times the *j*_*th*_ permutation occurs. The embedding dimensions $m=3,4,\ldots ,7$ have been suggested, and we selected *m* = 6 to extract most of the information.

### Statistical analysis

Based on a previous study^9^, the sample size was calculated using power analysis to detect a mean difference of 19 in the BIS value and a standard deviation of 20. Eighteen patients in each group were estimated to provide 80% power and a Type I error of 0.05.

Clinical characteristics were described as mean (standard deviation) or median [IQR] as appropriate. All variables were tested for normality using the Kolmogorov-Smirnov test. Continuous variables were compared using the Student’s t-test or Mann-Whitney test. For categorical variables, Fisher’s exact test and chi-square test were used. Repeated measures analysis of variance was used to evaluate the interaction of time and mean arterial pressure (MAP) and heart rate (HR) between the propofol and thiopental groups.

Analyses for spectral data were performed using a custom-written MATLAB code. We used a Wilcoxon rank-sum test to compare spectral estimates between different stages (Stage I vs Stage II vs Stage III) and between the two groups. A *p*-value < 0.05 was considered significant. For comparisons with awake data, we computed 95% confidence intervals (CI) using a bootstrapping algorithm included in the Chronux toolbox^19^. As the number of awake samples was small (n = 8), we drew bootstrap samples from the spectra with replacement across frequencies. We then calculated the difference between the spectra. We repeated this process 10,000 times and calculated the 95% CI for the median difference between the two groups at each frequency.

## Results

### Study population

**Figure 1:**
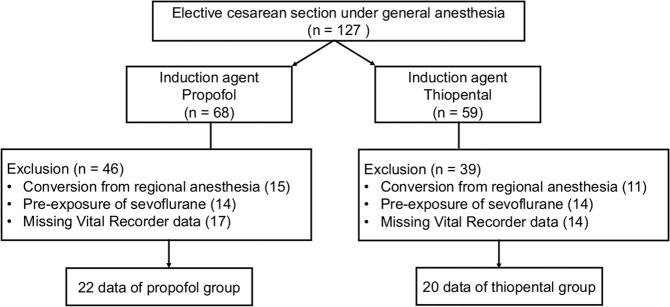
Patients flow diagram.

There were 127 cases of elective cesarean section under general anesthesia between September 2017 and May 2019 at our institute. The propofol and thiopental groups included 68 (53.5%) and 59 patients (46.5%), respectively. A total of 85 cases were excluded because there had been a failure of regional anesthesia (n = 26), missing Vital Recorder data (n = 31), or there was pre-exposure to sevoflurane at the pre-oxygenation stage (n = 28). The flow chart of the study population is shown in Fig. 1. We analyzed 42 patients who had an elective cesarean section under general anesthesia, 22 in the propofol and 20 in the thiopental group. There were no significant demographic differences between the two groups except in their respective body mass indexes. (Table 1). There were no intubation difficulties reported. No events were recorded for intraoperative awareness after the surgery in the study population.

**Table 1:** Demographic data of thiopental and propofol groups.

	Thiopental (n = 20)	Propofol (n = 22)
Age (yr)	36.0 ± 5.1	34.8 ± 3.7
Height (cm)	162.0 [159.0; 163.3]	162.4 [157.0; 166.0]
Weight (kg)	68.2 ± 11.0	72.4 ± 11.8
BMI (kg/m^2^)*	25.9 ± 4.1	28.7 ± 4.6
Gestational age (weeks)	38 [37.0; 38.0]	37.0 [36.0; 37.0]
**Indications for general anesthesia**		
Placenta previa totalis	11 (55)	14 (63.6)
Others	9 (45)	8 (36.4)
Patients wanted	3	4
Spinal surgery	3	3
Previous open abdominal surgery	3	1
Induction agent dose (mg)	337.5 [300; 350]	140.0 [130.0; 160.0]
Time from induction to Stage II (s)	114.6 ± 19.8	118.9 ± 29.3
Time from induction to delivery (s)	457.0 ± 79.0	387.8 ± 97.2

Data are expressed as mean ± standard deviation or median with IQR or number (%). BMI, body mass index. Stage II, intubation completion. *p < 0.05 between two groups.

### BIS and EEG analysis

**Figure 2:**
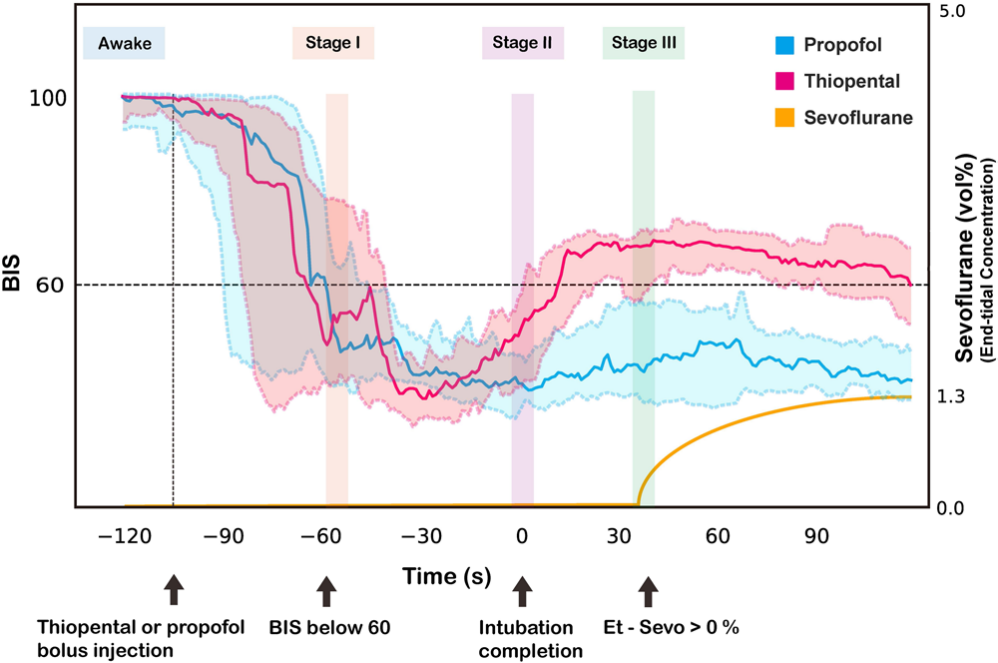
Experiment timeline: the time course of continuous BIS value and end-tidal sevoflurane concentration during the induction phase of general anesthesia in the thiopental and propofol groups. The solid line represents the median BIS value, and the shaded area represents its interquartile range. The vertical line shows each Stage analyzed segments: Awake, the first period with BIS below 60 (Stage I), the completion of intubation (Stage II), and the first period with end-tidal (Et) - Sevoflurane above 0 vol% (Stage III).

Figure 2 shows continuous BIS values (median [IQR]) from anesthesia induction until neonate delivery in each group. After Stage II, the median BIS value in the thiopental group increased above 60 and remained high, while the propofol group maintained a median BIS value lower than 60 through Stages II and III. In Stage III, there was a significant difference in the median BIS values between the thiopental and the propofol groups (67.9 [18.66] vs. 44.5 [20.63], respectively, *p* = 0.002).

**Figure 3:**
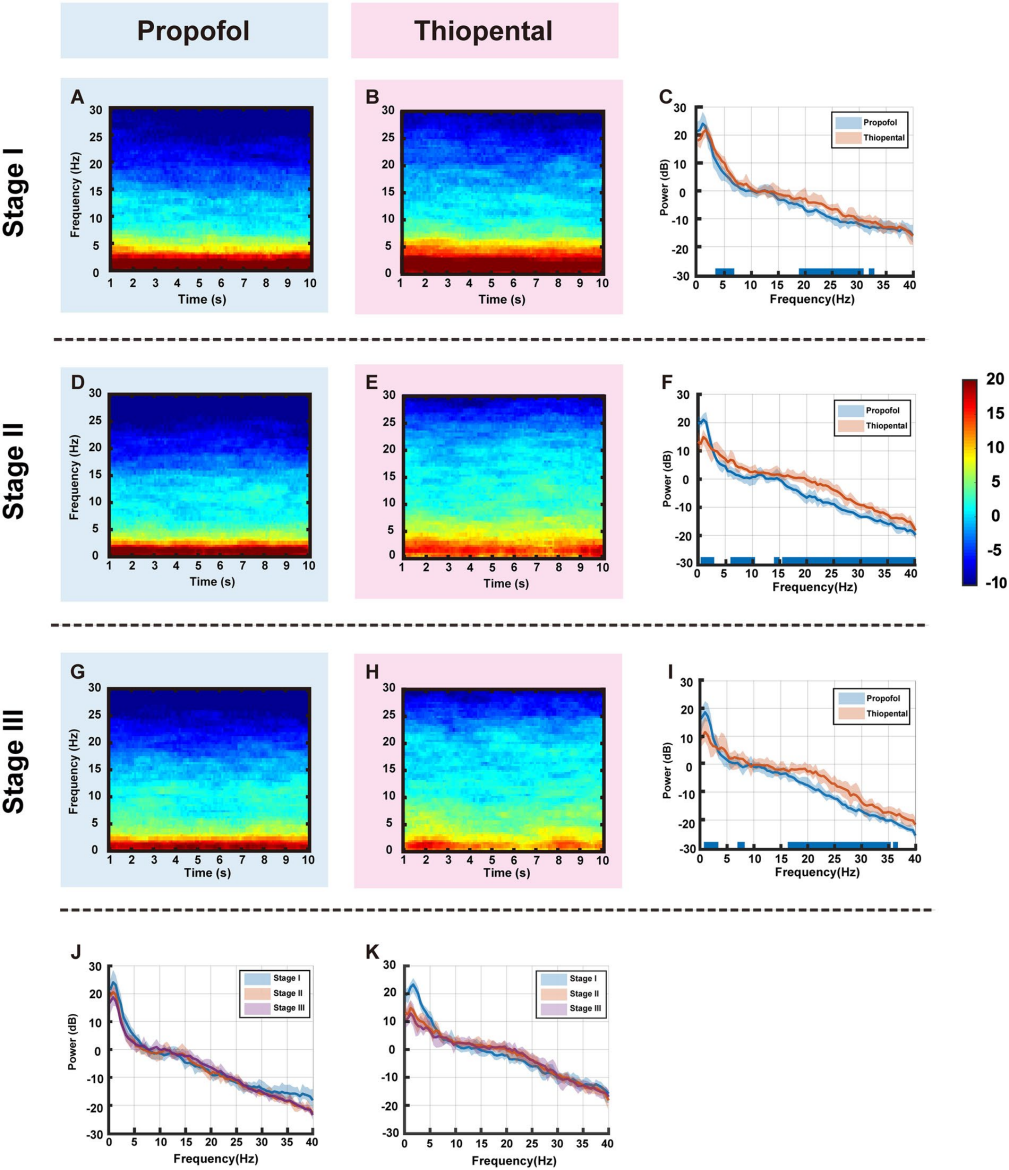
Representative group-median frontal spectrograms across 0.1 to 30 Hz at (**A,B**) Stage I, (**D,E**) II, and (**G,H**) III in the propofol and thiopental groups, respectively. The comparisons of group-median power spectra with an interquartile range interval (solid line, median) between the groups at (**C**) Stage I, (**F**) II, and (**I**) III. The blue overlay of the frequency axis represents frequencies with significant differences between the two spectra (*p* < 0.05). The comparisons of group-median power spectra with an interquartile range interval (solid line, median) over stages in the (**J**) propofol and (**K**) thiopental groups.

Group-median spectrograms were computed (Fig. 3A–H), and group level median spectra with IQR interval are presented in Fig. 3C,F,I–K. We found that the spectrogram of the thiopental group had decreased slow-delta (0.1–4 Hz) oscillations in Stage II and III compared to the propofol group. For a detailed comparison, we evaluated the power spectrum differences between the propofol- and thiopental-induced brain states across all stages. In Stage I, the thiopental group showed increased theta (4–6.5 Hz) and beta oscillations (19–30.5 Hz, 31.5–32.5 Hz) compared to the propofol group (Fig. 3C, *p* < 0.05). We noticed that slow-delta (0.1–3.5 Hz) oscillations were not significantly different in the two groups (*p* > 0.05). Stage II showed a dramatic decrease in slow-delta (0.1–3.5 Hz) power and an increase in theta-alpha (6–10 Hz) and beta (14–40 Hz) power in the thiopental group compared to the propofol group (Fig. 3D–F, *p* < 0.05). In Stage III, the thiopental group still had lower slow-delta (0.5–4 Hz) oscillations and larger beta (16–35 Hz) oscillations than the propofol group (Fig. 3G–I). In the propofol group, the median slow-delta (0.1–4 Hz) peak power decreased by 3.59 dB and 1.73 dB from Stage I to Stage II and from Stage II to Stage III, respectively (*p* < 0.05, Fig. 3J), while the thiopental group showed a more significant decrease in the slow-delta peak power from Stage I to Stage II (8.29 dB, *p* < 0.05, Fig. 3K).

**Figure 4:**
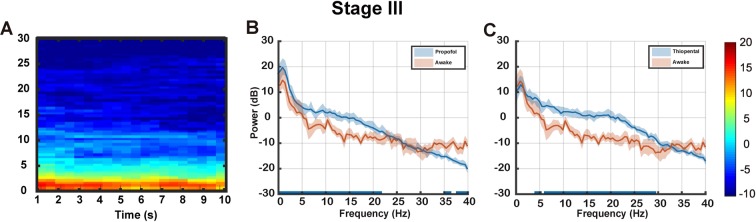
Awake analysis. (**A**) Group-median spectrogram while awake (n = 8). Group-median spectral power comparisons with interquartile range interval (solid line, median) between awake and Stage III in the (**B**) propofol group and (**C**) thiopental group. The blue overlay of the frequency axis represents frequencies with significant differences between the two spectra (*p* < 0.05).

For this retrospective study, it was difficult to find clean EEG segments recorded when the patients were awake. The eight patients with artifact-free EEGs in their awake states were selected by visual inspection. We computed a group-median spectrogram for the awake state (n = 8) (Fig. 4A). We then evaluated the power spectra differences between Stage III and the awake state in both the propofol and thiopental groups. Compared to the awake state, the thiopental group at Stage III had no significant difference in slow-delta (0.1–4 Hz) oscillations (Fig. 4C) while the propofol group had significantly larger slow-delta, theta, alpha and lower beta (0.1–22 Hz) oscillations (*p* < 0.05, Fig. 4B).

**Figure 5:**
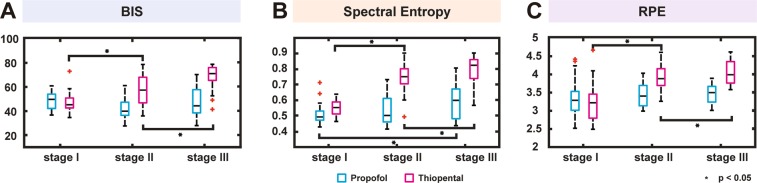
Entropy analysis. The comparisons of (**A**) BIS, (**B**) spectral entropy and (**C**) Renyi permutation entropy (RPE) between the propofol and thiopental groups at each stage. *p < 0.05, + and ‡ outlier value.

To assess the risk of awareness, we compared BIS, spectral entropy, and RPE in the propofol and thiopental groups across all stages (Fig. 5A–C). Spectral entropy and RPE were calculated using raw EEG extracted from the BIS monitor. In the thiopental group, BIS, spectral entropy, and RPE significantly increased when shifting from Stage I to II, and Stage II and III (*p* < 0.05, Fig. 5A–C). The propofol group did not have significant increases in BIS or RPE when moving between stages, but the spectral entropy was significantly different in Stage I and III (*p* < 0.05, Fig. 5B). The median spectral entropy in the propofol group (0.60) was much smaller than that in the thiopental group (0.83) in Stage III.

### Comparing hemodynamic variables between the propofol and thiopental groups

**Figure 6:**
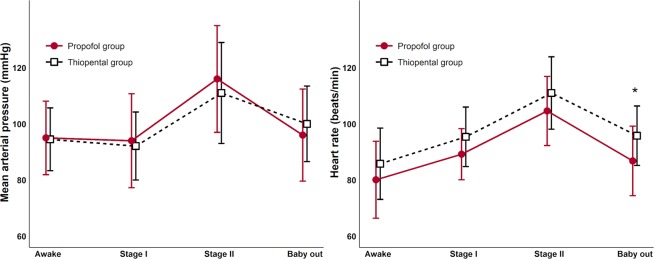
Mean arterial pressure and heart rate graph in propofol and thiopental group at each event point. *p < 0.05 vs thiopental group.

All maternal MAP and HR were compared at each event time-point (awake, Stage I, II and neonate delivery, Fig. 6). Although the change of MAP and HR within each group was significant between each event time-point, there was no significant interaction between the group and event time-point in MAP and HR (*p* = 0.190 and 0.756, respectively).

## Discussion

This EEG spectral analysis demonstrates that the thiopental group was associated with lighter anesthesia when RSI was used during cesarean section. The thiopental group experienced a more significant decrease in slow-delta wave activity and an increase of beta activity after intubation before neonatal delivery compared to the propofol group. This was similar to awake-state EEG oscillations. Spectral entropy and RPE also significantly increased after intubation in the thiopental group. Our findings suggest that the possibility of intraoperative awareness may be higher with thiopental-induced general anesthesia in pregnant women. It was in line with the results of the NAP5 study, which showed that the use of thiopental for RSI was a risk factor of awareness during anesthesia^3^.

Intravenous induction agents are important for maintaining an adequate depth of general anesthesia for cesarean section until volatile anesthetics reach a sufficient concentration in the brain^3^, since the use of supplementary opioids and other sedatives are avoided before neonatal deliveries^20^. During this interval, the risk of maternal awareness may be increased. After the injection of an intravenous agent, anesthetic depth rapidly declines until the brain concentration of the inhalation agents increases. This is supported in our study by showing that the median slow-delta peak power in both groups is lower in Stage II and III compared to than in Stage I. In Stage II and III, the thiopental group had a more significant reduction in median slow-delta power than the propofol group. If the dose of thiopental was increased, stable anesthetic depth during the general anesthesia induction period might be achieved. However, it is difficult to increase the dose of the induction agent due to concerns for the neonate’s safety. Propofol can maintain a stable BIS level and spectral analysis power in most patients, which means 2 mg/kg of propofol may supply adequate anesthetic depth during general anesthesia induction for cesarean section.

We analyzed awareness risk using the spectral entropy and RPE indexes, which were computed using raw EEG data extracted from the BIS monitor. The exact algorithm for BIS has not been released, but it is known that BIS is calculated from three factors: (1) degree to which EEG oscillations are in phase (bicoherence); (2) amount of EEG power in the delta (1–4 Hz) versus beta (13–30 Hz) range (power spectrum); and (3) proportion of the EEG that is isoelectric^21^. Using the combination of factors, the BIS monitor compares the recorded EEG signal with a previous database to produce a number that reflects the level of hypnosis. However, entropy reflects the irregularities in the EEG signal, which is independent of the amplitude scales of the EEG signal that are patient dependent. The main idea is that increasing depth of anesthesia causes an increase in regularity of the EEG signal and consequently, the decrease in entropy. In this regard, BIS and entropies may have different trends for the depth of general anesthesia^22^. Therefore, showing entropy indexes and BIS together could aid in the monitoring of intraoperative awareness during cesarean section. In our results, BIS, spectral entropy and RPE have the same trend over Stages I, II, and III, which indicates clearly that thiopental-induced general anesthesia has higher awareness risk compared to propofol-induced anesthesia.

In a study of non-parturients, a bolus of thiopental 4 mg/kg and propofol 2 mg/kg was administered, the BIS value was monitored, and the isolated forearm technique was employed^23^. The results of the study showed that the recovery time to consciousness was faster for thiopental than for propofol, which was due to the differences in the pharmacodynamic and pharmacokinetic properties of the two drugs. Though thiopental (a barbiturate) and propofol (an alkylphenol) have different chemical structures, they have the same site of action, the gamma-aminobutyric acid (GABA_A_) receptors. When given a single intravenous dose of these agents, they are rapidly redistributed from the central to maternal peripheral tissue as well as to the placenta^24^. While both drugs cause changes to the regional cerebral blood flow (rCBF), they do so at different locations in the brain. Thiopental decreases the rCBF in the cerebellar and posterior brain areas, whereas propofol affects the frontal area^25^. Recent studies have reported that the continuous infusion of propofol disrupts functional relationships between the frontal cortex and the thalamus^26,27^. The pharmacokinetic differences may also have been influenced by the effects of pregnancy on the phramacokinetic actions of drugs. In the case of thiopental, pregnant women have an increased volume of distribution and more rapid clearance because of decreases in the elimination half-life. However, it does not require a dose increase^28^. Although propofol pharmacokinetics have been well characterized in adult populations, little is known about it during pregnancy^29^. The effect-site concentration of both induction agents in our study may play an important role, but there are relatively few studies comparing thiopental and propofol pharmacokinetics during pregnancy. The detailed differences need to be further investigated.

Currently, there is no optimal anesthetic management for cesarean section. Our management for general anesthesia for pregnant women may be outdated. Recently, several recommendations and evidences to avoid maternal intraoperative awareness during general anesthesia have been presented. An additional dose of an induction agent given immediately before endotracheal intubation or opioid-based anesthesia could help maintain a stable depth of anesthesia^30^. Increasing evidence indicates that remifentanil administration at the induction of general anesthesia is safe for both the mother and the baby^31–33^. Pre-exposure of sevoflurane would reduce the BIS index during the interval before delivery^8^. An end-tidal concentration of sevoflurane 1.2–1.5 vol% with 50% N_2_O would be required to maintain BIS values less than 60^34^, which is known to suppress uterine contraction by about only 30%^35^. Ueyama *et al*.^36^ reported that pregnant women does not have a lower minimal alveolar concentration (MAC) compared to non-pregnant women. Although these results would need to be further investigated, it implies the need for modifying conventional general anesthesia management for cesarean section. The updated method should guarantee the safety of the parturient as well as of the neonate.

One of the main limitations of this study is that it was retrospective, and patients could not be randomized to receive each induction agent. Prospective studies using pregnant women generally require considerable effort due to many safety and ethical issues in studying this population. Further prospective studies would need to evaluate whether a specific agent reduces the risk of intraoperative awareness. Secondly, the reliability of BIS monitoring, especially during RSI, might be low. Time delays have been observed to calculate the BIS index at the transition of different anesthetic states^37^. After a bolus injection of propofol or thiopental for RSI, BIS values become unusually low when large delta wave was transiently emerging regardless of hypnotic level^38^. Zand *et al*.^39^ reported that isolated forearm test responses were seen in 46% of the parturients at intubation, and even mean BIS values were lower than 60. If adequate analgesic drugs were used during RSI, it might be influenced EEG parameters^40^. Further, the BIS responds to neuromuscular blocking drugs (NMBD)^41,42^, and there is a possibility that the patient recovers from consciousness during the RSI period even when the BIS value is low, since NMBD would be effective during that time. Succinylcholine may also interfere with interpretations. Succinylcholine causes fasciculation and leads to raised EMG, which may influence the EEG signal and BIS values. However, we intubated after the EMG in the BIS monitor fell to zero and immediately injected rocuronium following intubation. We therefore believe that the EEG differences between groups were unaffected. In addition, the BIS sensor is affected by multiple factors that may interfere with EEG interpretation. Our raw EEG data included segments with artefacts and noise caused by the physical stimulation of patients such as intubation, electrocautery, and traction of the abdominal wall by the surgeon. Therefore, we analyzed the 10 second artifact-free EEG segments. Thirdly, the depth of anesthesia during cesarean section was the result of different combinations of drugs, including intravenous hypnotics, volatile anesthetics and N_2_O. There is a possibility that N_2_O may influence EEG dynamics. It suppresses low-frequency (delta and theta) EEG power and consequently pronounces high-frequency oscillations^43^, which may affect BIS monitoring. Both groups were given N_2_O in Stage III, but the intervals included in our EEG analysis were short. Thus, we feel assured that the effect of N_2_O on the results is minimal. Fourthly, if bilateral and multiple-channel EEG monitors could be used for this type of study, we may acquire more detailed information such as a coherence analysis. Finally, this study briefly assessed the patient’s unpleasant dreaming and any other experiences related to awareness after cesarean section, and there were no complaints nor other specific reports from patients related to intraoperative awareness. This assessment which dependent on the patient’s memory and experiences, cannot detect the exact occurrence of intraoperative awareness. Due to the administration of midazolam and opioid after neonate delivery, current parturients seemed to have no experience related to awareness. Therefore, it may imply that a need for structural questionnaires for patients who are at high risk for intraoperative awareness during general anesthesia.

In conclusion, this frontal spectral EEG analysis demonstrates that propofol for RSI in cesarean section maintained a more adequate anesthetic depth than the thiopental. Thiopental induction may increase intraoperative awareness risk in entropy analysis.
